# Water Content Variations and Pepper Water-Use Efficiency of Yunnan Laterite Under Root-Zone Micro-Irrigation

**DOI:** 10.3389/fpls.2022.918288

**Published:** 2022-07-05

**Authors:** Yijie Zhang, Zhenjie Yang, Lingqiong Kong, Haolin Yang, Yang Yu, Yuqing Zhao

**Affiliations:** ^1^College of Mechanical and Electrical Engineering, Yunnan Agricultural University, Kunming, China; ^2^College of Water Resources and Hydraulic Engineering, Yunnan Agricultural University, Kunming, China; ^3^College of Engineering, Nanjing Agricultural University, Nanjing, China; ^4^The Key Laboratory for Crop Production and Smart Agriculture of Yunnan Province, Yunnan Agricultural University, Kunming, China

**Keywords:** root-zone micro-irrigation, Yunnan laterite, soil water content variation, hot pepper, water use efficiency (WUE)

## Abstract

Understanding the water content variations in Yunnan laterite (red loam soil, SR) in small-scale environments and exploring the potential for crop water-use efficiency (WUE) improvement are crucial for improving water-saving irrigation technologies used in greenhouse agriculture in Yunnan, China. In this study, a closed-loop model for calculating soil water in greenhouse potted cultivation was established based on water conservation. A Yunnan SR, yellow sand soil (SY), and a 1:1 SR–SY mixture (SM) subjected to root-zone micro-irrigation or surface-drip irrigation were experimentally examined to compare their water content variations and pepper WUEs. The results showed that the soil type and soil type–irrigation mode interaction had significant effects on both soil evaporation and pepper WUE, and that the variations in soil evaporation with respect to time can be expressed using a cubic polynomial function. In small-scale greenhouse cultivation, IG has good water-saving potential and is suitable for the SR (which has a better water-retention capacity), whereas IM is more suitable for the SY (which has a better water-penetration capacity). Mixing certain proportions of the SY into the SR will effectively impact the water content variations and crop WUE and provide opportunities for further improving the water-saving efficiency.

## Introduction

Subsurface irrigation is a high-efficiency water-saving irrigation technique with remarkable potential for improving agricultural water productivity (Du et al., 2017). A significant number of studies have investigated the effects of subsurface irrigation on soil water content and crop growth for different types of soils and crops. For example, Ma et al. (2020) investigated the water-use efficiency (WUE) of grapes grown on loamy sand in a semi-arid region of Washington state, United States, under direct root-zone irrigation. They observed that the grape WUE was improved by 9–11% compared with that under surface-drip irrigation. Martinez and Reca (2014) investigated the use of a new subsurface drip irrigation (SSDI) method for olive cultivation on calcareous soil in southern Spain. In the method, emitters were installed on the soil surface to discharge water into perforated plastic pipes that were vertically inserted into the ground. The method improved the olive yield by 8.3% and reduced the water consumption by 20% compared with those under surface-drip irrigation. Jiao et al. (2020) investigated the WUE of jujube grown on sierozem soil in an arid region of central Ningxia, China, under subsurface infiltration irrigation. They observed that as the buried depth of the irrigation pipe and the irrigation amount increased, the soil water content in both the vertical and horizontal directions first increased and then decreased. Zhu et al. (2020) investigated the WUE of cherry radish grown in clay loam in Beijing, China, under negative-pressure subsurface irrigation. They observed that negative-pressure irrigation improved the biomass WUE by more than 50% compared with that under conventional irrigation.

In summary, the soil water content variations and crop water productivities obtained using different subsurface irrigation modes, soils, crops, and experimental methods were significantly different. Previous studies have investigated numerous different types of soils, including sandy loams, calcareous soils, and clay loams. However, relatively few studies have examined the water content variations in a unique red soil (SR), which is a lateritic soil, in Yunnan, China. There are even fewer studies on the water variations (quantitative variations in major factors such as soil water infiltration, moisture content, evaporation, and crop water consumption) in SR, either with or without crop cultivation, and on the WUEs of crops produced under root-zone micro-irrigation. Therefore, in the background of seasonal drought and engineered water scarcity in Yunnan, China, this paper from the perspective of water content variations in Yunnan red soil under root-zone micro-irrigation explores the key elements involved in the soil water content variations, explores the potential of WUE, and provides experimental data to support the effective application of root-zone micro-irrigation technology in the local.

Root-zone micro-irrigation, which is a subsurface irrigation technique, is a new, water-saving irrigation technique that can significantly improve crop yield, quality, and WUE (Zhang et al., 2019a; de Deus et al., 2020). This technique uses flexibly configured independent subsurface micro-emitters to transport water and liquid fertilizers in a pipe to the major active layers of the crop root zone in a small, uniform, targeted, and precise flow, thus effectively reducing surface evaporation. This technique has broad prospects for application in greenhouse cultivation owing to its high water-saving potential. However, Yu et al. (2015) observed that the SR has a high clay content and poor aeration capacity and thus constrains root respiration and WUE improvement. By contrast, yellow sand (SY) has a better aeration capacity because the irrigation water in SY is subjected mainly to gravitational potential and thus can be more easily infiltrated downward. Therefore, the hypothesis that crop WUE may be effectively improved via the mixing of a certain proportion of SY into SR needs to be experimentally investigated.

For the continuity of the study, based on the methods and findings of previous studies (Yu et al., 2018, Yu et al., 2015; Zhang et al., 2018, 2019a,b), this study first quantitatively examined the water content of a Yunnan SR at a small scale (flowerpot) under IG and in the absence of crop cultivation, and then the potential of IG for improving the WUE of a specific crop (Sichuan hot pepper) cultivated on the SR. Our results provide theoretical and experimental data support for the high-efficiency application of root-zone micro-irrigation in greenhouse agriculture in Yunnan, China.

## Materials and Methods

### Test Apparatus and Materials

#### Device for Precise Collection of Soil Water Infiltration

**FIGURE 1:**
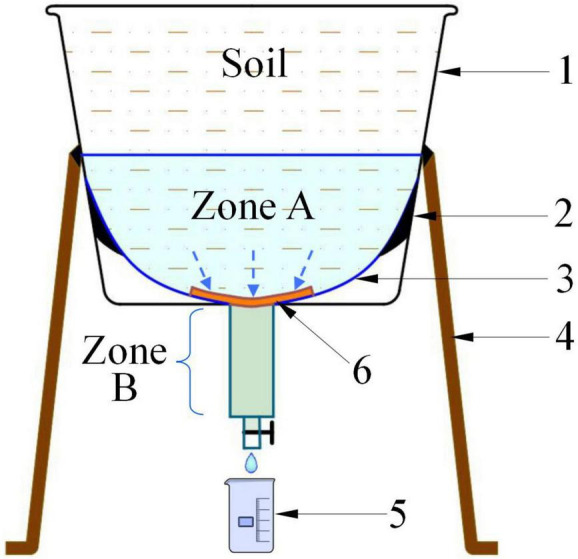
Device for collecting and measuring amount of water seepage under the soil. **(A)** Is the guiding area of water seepage. **(B)** Is the collection area of water seepage. 1. Outer barrel; 2. Sealant; 3. Inner barrel; 4. Supporting framework; 5. Measuring cup; 6. Gauze filter.

A device that was fabricated in-house was used for the closed-loop collection of downward infiltrated soil water (Figure 1). The device consisted mainly of an outer barrel (made from flower pot; outer diameter 34 cm; inner diameter 29 cm; height 23.5 cm), inner barrel (made from a larger funnel and the set inside the outer barrel; outer diameter 25.5 cm; inner diameter 25 cm, height 21 cm), measuring cup, supporting framework, sealant, and gauze filter (filter mesh 800; filtration accuracy 19 μm).

#### Test Materials

The following soils were tested: (1) SR; (2) SY; and (3) SR–SY, in a 1:1 uniform mixture (SM). The SR sample was collected in a greenhouse laboratory at Yunnan Agricultural University (102°45’5”E, 25°8’7”N), whereas the SY sample was procured from an external source, the SR was slightly compressed to make it the same bulk density as the SY. Table 1 shows the basic physical properties of the SR and SY. Both soils were naturally dried and screened using a 2 mm sieve for subsequent use.

**TABLE 1:** Physical properties of Yunnan laterite and yellow sand soil.

Soil type	Bulk density/(g⋅cm^–3^)	Field capacity/%	Soil particle composition/%		
			
			Clay (< 0.002 mm)	Silt (≥ 0.002 to 0.02 mm)	Sand (≥ 0.02 to 2 mm)
Yunnan red loam (SR)	1.23	22.3	39.47	35.32	25.21
Yellow sand (SY)	1.23	0.36	4.66	25.13	70.21

Emitter: A 1821A flow-adjustable emitter (Shanghai Huawei Water Saving Irrigation Corp., Ltd., Shanghai, China) was used. Its overall shape is that of a flat disk, with eight narrow openings (1 mm x 1.5 mm) on the circumferential side wall for the horizontal discharge of water. The flow rate was set to 2 L/h. This emitter is commonly used for micro-irrigation in Yunnan’s greenhouse cultivation.

Test crop: The Sichuan Laxiu 838 cultivar of hot pepper was cultivated in potted soil. The cultivar has a high yield throughout the year, an intermediate intensity of pungency, a beautiful berry morphology, and is suitable for potted cultivation.

#### Potted Pepper Cultivation and Greenhouse Environmental Conditions

First, seedlings of peppers at the 6–8 leaf stage (purchased from the Longtou Street Farmers’ Market in Kunming, Yunnan) were refined for a period of 2 weeks. Then, at the end of the seedling development, the pepper seedlings with similar height and stem thickness and good growth were transplanted into test pots, three plants in each pot, and 1 kg of farm organic fertilizer (fermented pig manure) was added to each pot in advance. After the start of the irrigation experiment, as little chemical fertilizers and pesticides were used as possible to exclude their effects on soil properties and soil water content measurement.

The experiment was conducted in a plastic greenhouse arch, where the temperature and humidity (average daytime temperature 28°C, average nighttime 16°C; average humidity 54%) and ventilation were regulated by means of roller shades and shade nets. No visible pests or diseases occurred during the whole experiment.

### Experimental Design

Two sets of tests were conducted in the greenhouse laboratory at Yunnan Agricultural University: (1) tests on soil water content variations (SWCT) with no crop cultivation; and (2) tests on the WUE (WUET) of the pepper in potted cultivation (Table 2).

**TABLE 2:** Experimental design of soil water content variations (SWCT) and tests on the water-use efficiency (WUET).

Test name	Irrigation type	Soil type	Irrigation frequency/days
SWCT	Root-zone micro-irrigation	SR SY SM	2, 4, 6
WUET	Surface drip irrigation		3

Each set of tests involved six combinations of treatment conditions (two irrigation modes × three soils), with three replicates per combination for the SWCT (i.e., a total of 18 tests), and six replicates per combination for the WUET (i.e., a total of 36 tests). In both sets of tests, the irrigation pressure was set to 0.1 MPa. The irrigation scheme used in this study was adopted from experiments conducted by Yu et al. (2015) and Zhang et al. (2019a).

**FIGURE 2:**
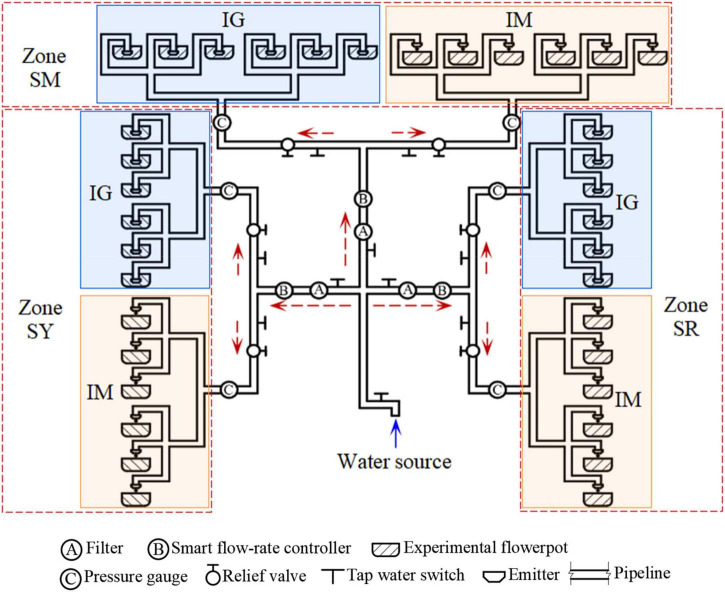
Schematic of WUET experimental system.

The two sets of tests were conducted using similar setups, both of which consisted mainly of a water source (tap water), PVC pipes, Y-100 pressure gages (Shanghai Yichuan Instrument Factory, Shanghai, China), ZL-LCD-M smart flow rate controllers (Foshan Shunde Zhongjiang Energy Saving Electronics Co., Ltd., Foshang, China), pressure-reducing valves with a pressure control range of 0–0.8 MPa (Nanjing Fangwei Valve Co., Ltd., Nanjing, China), emitters, and flowerpots. Both test setups consisted of three irrigation areas for the three soils (i.e., SR, SY, and SM). Each irrigation area consisted of two sub-areas for root-zone micro-irrigation (hereafter, represented by IG) and surface-drip irrigation (hereafter, represented by IM). In the SWCT, each irrigation area consisted of six branches (i.e., three branches each for IG and IM). In the WUET, each irrigation area consisted of 12 branches (i.e., six branches each for IG and IM). Figure 2 shows a schematic illustration of the setup for the WUET.

The main measurement instruments used in the tests included a time-domain reflectometry (TDR) SK-100 soil moisture meter (Shanghai Jingke Precision Instruments, Shanghai, China), a 330 thermostatic drying oven (Shanghai Yetuo Instruments Co., Ltd., Shanghai, China), an electronic scale with a precision of 0.001 g (Dongguan Nancheng Changxie Electronics Products Factory, Dongguan, China), and a 500 ml measuring cup with a precision of 0.5 ml (Jiangsu Jintan Huaou Experimental Apparatus Factory, Jintan, China).

### Experimental Methods

#### Calculation of Soil Water Content Correction Factor

The soil water content measurements obtained using a water moisture meter were corrected against measurements obtained using a weighing method to derive more accurate estimates of soil water content. The correction factor K for water content measurements of greenhouse potted soil obtained using the water moisture meter is calculated as


(1) $$K=\frac{\rho_{0}}{n}\,\sum_{i=1}^{n}\frac{\Delta \,S_{i}^{\prime }}{\Delta \,S_{i}}$$

where ΔSi is the volumetric variation in the soil water content in the time interval from the i-th irrigation to the i + 1-th irrigation (ΔTi) as obtained using the moisture meter (%), ΔS’i is the mass variation in the soil water content during time interval ΔTi as obtained using the weighing method (%), ρ_0_ is the correction factor for the volumetric water content against the mass water content, and n is the number of tests.

The moisture meter was used to measure the soil water content using a layer-by-layer multiple-point method (Ministry of Water Resources of the People’s Republic of China, 2015; Zhang et al., 2019a). The soil mass in a flowerpot was defined as consisting of three layers from the soil surface downward, with each layer measuring 10 cm in thickness, and with the soil water content in each layer assumed to be uniform. Water content measurements were obtained at five points uniformly distributed along the perimeter of a 10-cm-radius circle in the middle of each soil layer. The average of the five measurements was used as an approximate of the water content of that soil layer.

#### Calculation of Crop Water Consumption and Soil Water Variations

**FIGURE 3:**
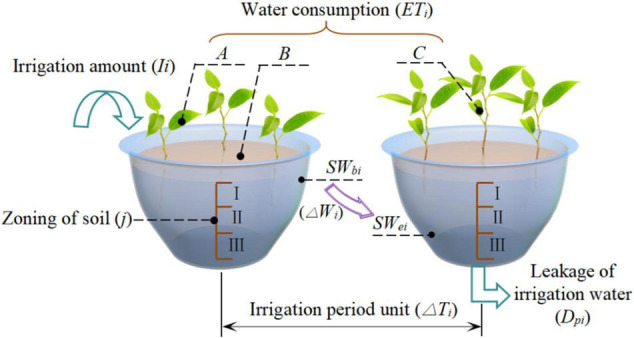
Schematic of test principle. The image shows the principle of water balance for the greenhouse potted crops within the research period (i.e., irrigation unit Δ*T_*i*_*). **(A–C)** represent crop transpiration, soil evaporation between crops, and volume of water contained within a plant, respectively. The crop water consumption (*ETi*) during the research period is the sum of A, B, and C. Symbols *SW*_*bi*_ and *SW*_*ei*_ represent the soil water content at the beginning and end of the research period, respectively. Thus, the change in soil water content during the research period is Δ*W_*i*_* = *SW*_*ei*_—*SW*_*bi*_. Labels I, II, and III represent the zoning (*j*) of the soil in the experimental flowerpot.

Figure 3 shows an illustration of the relationship between crop water consumption and soil water variations based on the principle of water conservation.

The relationship between crop water consumption and soil water variations during greenhouse potted cultivation, as shown in Figure 3, can be expressed as (Oweis et al., 2011; Zhang et al., 2019a; Wu et al., 2020).


(2) $$\sum_{i=1}^{n}E\,T_{i}=\sum_{i=1}^{n}I_{i}-\sum_{i=1}^{n}\Delta \,W_{i}-\sum_{i=1}^{n}D_{p\,i}$$

(3) $$\Delta \,W_{i}=\sum_{j=1}^{m}K\,V_{j}\,\Delta \,S_{i\,j}$$

where i is the number of irrigations applied during the test, j is the number of soil layers in the flowerpot, ETi is the crop water consumption during time interval ΔTi (soil water evaporation in the SWCT) (ml), Ii is the irrigation amount at the i-th irrigation (ml), ΔWi is the soil water content variation during time interval ΔTi (ml), Dpi is the amount of infiltrated water during time interval ΔTi (ml), K is the soil water content correction factor [see Equation (1)], Vj is the volume of the potted soil (cm^3^), and ΔSij is the water content variation in the j-th layer during time interval ΔTi as measured using the moisture meter (%). The SWCT included six combinations of treatment conditions (with three replicates for each combination) and involved 25 fixed-volume (500 ml per irrigation), variable-frequency (2 days for the 1st to 15th irrigations; 4 days for the 16th to 20th irrigations; 6 days for the 20th to 25th irrigations) irrigations for 80 days. In contrast, the WUET included six combinations of treatment conditions (with six replicates for each combination) and involved 19 fixed-volume (600 ml per irrigation), fixed-frequency (3 days) irrigations. In both sets of tests, the frequency of irrigation coincided with the frequency of measurement. The emitter was placed at the center of the flowerpot. For IM, the emitter was placed 5 cm above the soil surface, whereas for IG, the emitter was buried 7.5 cm below the soil surface.

#### Calculation of Water-Use Efficiency (WUE)

water-use efficiency refers to the amount of assimilated material produced by plants per unit of water consumption, which essentially reflects the relationship between plant water consumption and its dry matter production, and is a comprehensive physiological and ecological index to evaluate the suitability of plant growth (Wu et al., 2020). Based on the principles of the tests, the biomass WUE (WUEB) of the pepper cultivated in greenhouse potted soil can be calculated as (Zhang et al., 2019a; Wu et al., 2020; Zhu et al., 2020).


(4) $$W\,U\,E_{B\,n}=W_{B\,n}/\sum_{i=1}^{n}E\,T_{i}$$

where WUEBn is the pepper WUE obtained at the i-th destructive sampling (kg⋅m^–3^), WBn is the pepper dry biomass obtained at the i-th destructive sampling (kg), and ETi is defined in Equation (2). One flowerpot (with three plants, i.e., three replicates per flowerpot) was randomly sampled from each treatment at each destructive sampling. For destructive sampling (i.e., harvesting), first, the soil of the target pots was completely moistened with water; then, the three harvested samples in the pots were dug out with the roots, rinsed well, and blotted with paper to dry the adhering water; finally, the harvested samples were measured for fresh weight (weighed electronically) and dry weight (dried at constant temperature to constant weight, drying temperature 70, drying time about 2 days). The entire test involved a total of six (i.e., *n* = 6) destructive samplings, thereby obtaining the WUEs at six different growth stages of the pepper (spanning from the beginning of the test to the time of berry harvesting).

### Data Treatment

Multiple factor ANOVA was performed using the advanced version of the DPS v18.10 data treatment system (Tang, 2013). Multiple comparisons using Duncan method. The results of the data analysis were plotted using the SigmaPlot 14.0 software.

## Results and Analysis

### Statistical Significance of Effects of Irrigation Mode and Soil Type on Soil Evaporation in Absence of Crop Cultivation and on Crop Water-Use Efficiency (WUE)

An analysis of variance, with the irrigation mode and soil type as the independent variables and soil evaporation in the absence of crop cultivation and the pepper WUE as the dependent variables, was performed. The calculated p value is shown in Table 3.

**TABLE 3:** Variance analysis table.

Analysis Project Test category	Source of variation	Sum of squares	Degree of freedom	Mean square	*F* value	*P*-value
Experiment on influencing factors of crop-free soil evapotranspiration	Irrigation types (IT)	98380.130	1	98380.130	17.446	0.001**
	Soil types (ST)	21006493.500	2	10503246.750	1862.579	0.000***
	IT × ST	241005.518	2	120502.759	21.369	0.000***
Experiment on influencing factors of water-use efficiency of crops (pepper)	Irrigation types (IT)	0.000	1	0.000	0.000	0.983
	Soil types (ST)	2.519	2	1.260	6.047	0.006**
	IT × ST	1.417	2	0.709	3.402	0.047*

*, **, *** means P < 0.05, P < 0.01, P < 0.001 separately.

The results presented in Table 3 revealed the following: (1) In the absence of crop cultivation, the irrigation mode, soil type, and irrigation mode–soil type interaction affected the soil evaporation very significantly (*P* < 0.01 or *P* < 0.001). (2) During crop (pepper) cultivation, the soil type affected the pepper WUE very significantly (*P* < 0.01), whereas the irrigation mode–soil type interaction affected the pepper WUE significantly (*P* < 0.05). In contrast, the irrigation mode did not have any significant effect on the pepper WUE (*P* > 0.05).

### Water Content Variations in SR Under IG and Without Crop Cultivation

#### Effects of Irrigation Mode and Soil Type on Soil Water Content and Infiltration

**FIGURE 4:**
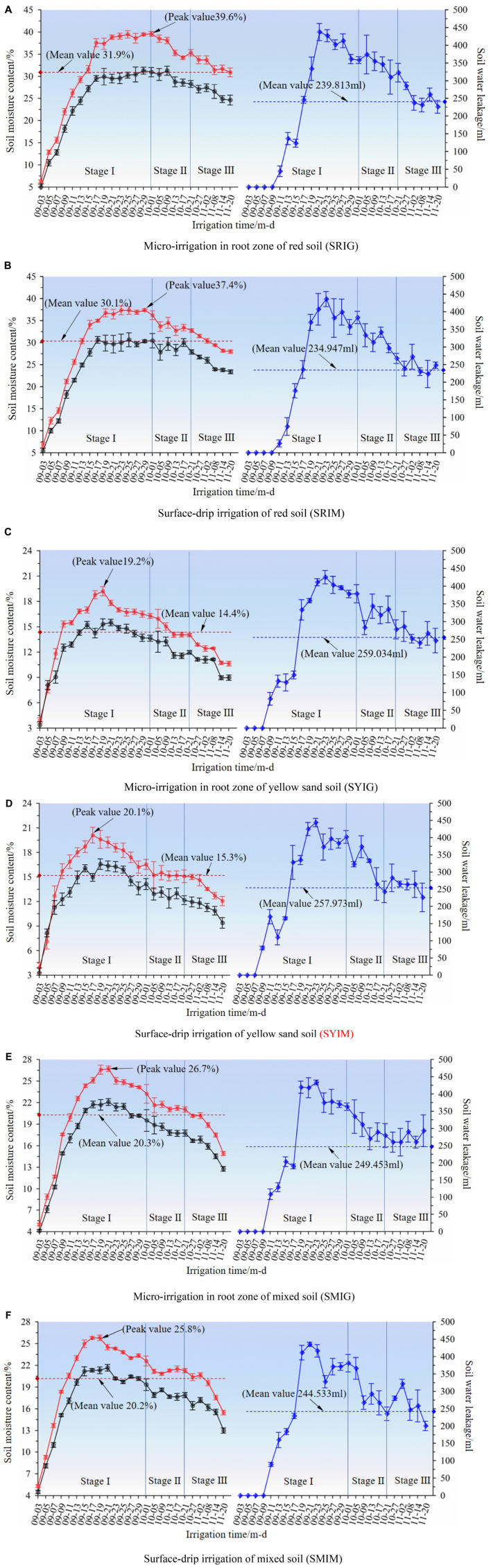
Variation map of moisture content (TDR and weighing method) and water drainage of non-crop soil against time. 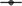 represents moisture meter method,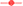 represents weighing method,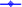 represents amount of soil water leakage. Stages I, II, and III represents three time-interval gradients, i.e., 2, 4, and 6 days, respectively.

The water content of the crop-free soil determined by the “moisture meter method” and “weighing method” for each treatment and the corresponding water leakage were plotted (Figure 4).

a. Micro-irrigation in root zone of red soil (SRIG)

b. Surface-drip irrigation of red soil (SRIM)

c. Micro-irrigation in root zone of yellow sand soil (SYIG)

d. Surface-drip irrigation of yellow sand soil (SYIM)

e. Micro-irrigation in root zone of mixed soil (SMIG)

f. Surface-drip irrigation of mixed soil (SMIM)

As shown Figures 4A–F, the soil water content (red line) and infiltration amount (blue line) in all the treatments varied in consistent trends—first increasing rapidly and then decreasing gradually. In stage I, the soil water content and infiltration for all combinations of treatment conditions increased rapidly and peaked. However, the times of peaking were different among the treatment conditions. Peaking occurred the earliest for SY. In contrast, peaking occurred the latest for SR treatments. From the peaking point to stage II, the soil water content levels for SRIG and SRIM were sustained close to the peak (39.6 and 37.4%, respectively) for more than half of the time interval (Figures 4A–B), indicating a good water-retention capacity. On the other hand, for SY and SM, the soil water content decreased rapidly from the peaking point to stage III. More specifically, the soil water content for the SYIG treatment conditions decreased by the largest magnitude—from 19.2% at the peak to 17.2%.

Further analysis showed that soil type had a very significant effect on the soil water content. This was demonstrated by the average water content levels for the tested treatment conditions—the average water content for SRIG (SRIM) was higher than that for SYIG (SYIM) by 121.7% (96.5%) on average and higher than that for SMIG (SMIM) by 56.7% (48.2%) on average. For a given soil, IG and IM result in insignificant differences in terms of soil water content. More specifically, the water content for SRIG was higher than that for SRIM by approximately 5.9%, the water content for SYIG was lower than that for SYIM by approximately 6.2%, whereas the water content for SMIG did not differ from that for SMIM (higher less than 0.1%).

The correction coefficient of the water content measurements for the different combinations of treatment conditions, as obtained using the moisture meter, were calculated using the test data and Equation (1) (Table 4).

**TABLE 4:** Correction coefficient K value table of soil moisture content measured.

Soil types Irrigation types	Yunnan red loam (SR)	Yellow sand (SY)	SR–SY, in a 1:1 uniform mixture (SM)
Root zone micro-irrigation (IG)	*K*_*SRIG*_ = 1.243	*K*_*SYIG*_ = 1.177	*K*_*SMIG*_ = 1.188
Surface drip irrigation (IM)	*K*_*SRIM*_ = 1.198	*K*_*SYIM*_ = 1.192	*K*_*SMIM*_ = 1.186

*The correction coefficient K value listed in the table are the average values of the ratio of soil moisture content measured by the “weighing method” and the “moisture meter method” under each treatment.*

The average volumetric water content measurements for the potted soil at the various pepper growth stages as obtained using the moisture meter were lower than the corresponding values obtained using the weighing method. For a given irrigation mode, the correction factor for the SR is larger than those for the two other soils. The aforementioned results indicate that the soil water content measurements obtained using the moisture meter (TDR SK-100) were lower than the actual values (Yang et al., 2017). In addition, the SR requires a larger correction factor because it has a high content of clay, which strongly affects the energy dissipation of the TDR electromagnetic waves and easily leads to the fuzzy reflection of these waves and, therefore, inaccurate measurements ([Bibr B5][Bibr B21]). The correction coefficient shown in Table 4 also provide data support for improving the accuracy of pepper WUE measurements in subsequent research.

#### Effects of Irrigation Mode and Soil Type on Soil Evaporation

**FIGURE 5:**
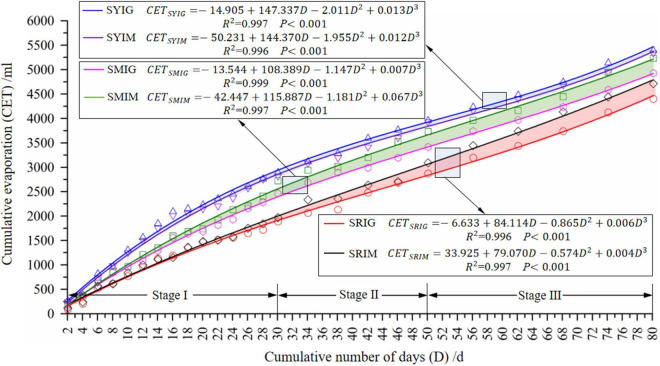
Variation in non-crop soil evaporation with time. Treatment condition codes SRIG, SRIM, SYIG, SYIM, SMIG, and SMIM indicate red soil subjected to root-zone micro-irrigation, red soil subjected to surface-drip irrigation, yellow sand soil subjected to root-zone micro-irrigation, yellow sand soil subjected to surface-drip irrigation, mixed soil subjected to root-zone micro-irrigation, and mixed soil subjected to surface-drip irrigation, respectively.

To further investigate the soil water content variations under IG and in the absence of crop cultivation, the amounts of cumulative soil evaporation (hereafter referred to as “soil evaporation”) at different time points were calculated using the measurement data and Equation (2). The variations in soil evaporation with respect to time were regressed using the non-linear least-squares method (Marquardt method). Several functions were tested and compared in the regression analysis. Finally, on the basis of the highest coefficient of correlation, one of the polynomial functions was selected to express the variations in soil evaporation with respect to time (Figure 5).

The results showed the following: (1) The soil cumulative evaporation with respect to time for all combinations of treatment conditions can be expressed using a cubic polynomial function with a high coefficient of correlation (with the crucial R^2^ value higher than 0.99). (2) The soil cumulative evaporation increased with respect to time for all combinations of treatment conditions, but with different trends of increase. More specifically, the rate of increase was the largest for SY, followed by SM and SR. In addition, the differences were affected by irrigation frequency. For example, in stage I, the difference between the average evaporation amount for SY and that for SR (SM) increased from 77.315 (59.627) ml to a peak of 946.981 (418.393) ml. In stages II and III, the difference decreased to 829.391 (230.402) ml. That is, the difference first increased and then decreased. Under high-frequency irrigation, the SY had the most active soil evaporation. As the frequency of irrigation decreased, the difference in water activity between the three soils decreased. (3) For a given soil, different irrigation modes led to different amounts of evaporation. More specifically, for SR and SM, IG led to lower evaporation than that for IM, with the difference increasing with time. For the SY, IG led to a slightly higher amount of evaporation than that for IM, with the difference remaining stable with time.

### Pepper Water-Use Efficiency (WUE) Under Root-Zone Micro-Irrigation (IG)

#### Water Consumption by Pepper Crop

**FIGURE 6:**
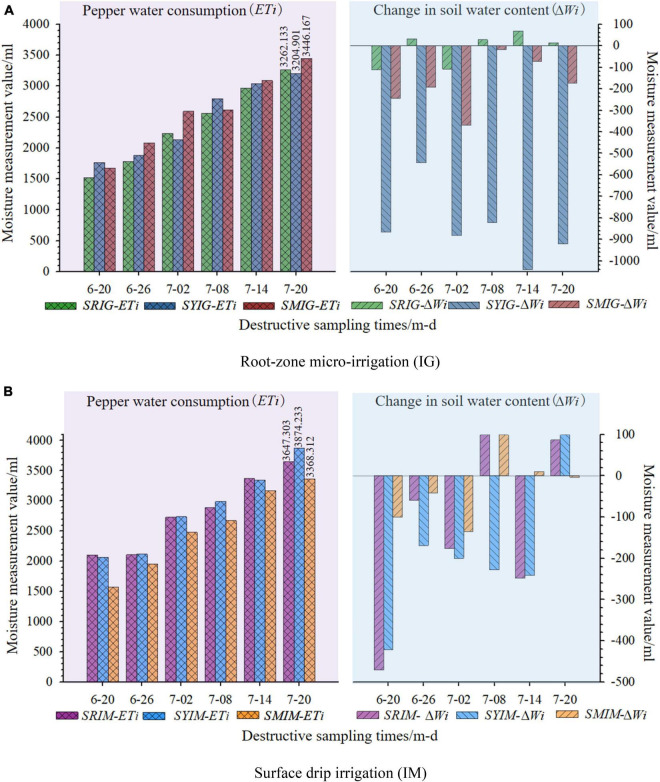
Water consumption and soil water variation for pepper crop. The meaning of the “treatment condition codes SRIG, SRIM, SYIG, SYIM, SMIG and SMIM” is detailed in the notes with Figure 5. **(A)** Root-zone micro-irrigation (IG); **(B)** surface drip irrigation (IM).

Figure 6 shows stacked graphs for the amounts of water consumption by the pepper crop under IG as obtained from six destructive samplings and the corresponding soil water content variations, compared with the corresponding values obtained under IM.

a. Root-zone micro-irrigation (IG)

b. Surface drip irrigation (IM)

The results revealed the following: (1) The water consumptions (ETi) of the pepper crop cultivated on the SR and SY under IG were lower than that under IM. The values of ETi for the SM under IG were approximately equal to those under IM. For example, at the sixth destructive sampling (see the values on the bar at 7–20 in Figure 6), the cumulative ETi of the pepper crop cultivated on the SR (SY) under IG (Figure 6A) was lower than that under IM (Figure 6B) by approximately 10.6% (17.3%), whereas the ETi of the pepper crop cultivated on the SM under IG was higher than that under IM by approximately 2.3%. (2) The absolute means (AMs) of the soil water content variations (ΔWi) for the six destructive samplings from the SR subjected to IG and IM were 60.167and 196.833 ml, respectively; that is, IG reduced the AM by 69.4% relative to that for IM. By comparison, the AMs for the SY and SM under IG were 847.024and 178.333 ml, respectively, which were significantly larger than those under IM (230.833and 68.833 ml, respectively), increasing by approximately 2.67- and 1.59-fold, respectively.

#### Pepper Water-Use Efficiency (WUE)

Table 5 shows the water consumptions, dry weights, and WUEs of the pepper crop for the sixth destructive sampling (i.e., at the time of berry harvesting) under the different treatment conditions.

**TABLE 5:** Water-use efficiency (WUE) under different treatment conditions at harvesting.

Treatment	Water consumption/g	Dry weight/g	WUE/(kg⋅m^–3^)	Relative change in rate of WUE (IG relative to IM)
SRIG	3262.133 ± 13.959*eE*	16.067 ± 1.553*aA*	4.924 ± 0.456*aA*	26.7%
SRIM	3647.300 ± 6.359*bB*	14.167 ± 2.074*abAB*	3.885 ± 0.576*bAB*	
SYIG	3204.901 ± 19.509*fF*	7.967 ± 2.804*dC*	2.489 ± 0.893*cB*	–17.1%
SYIM	3874.233 ± 9.827*aA*	11.633 ± 0.709*bcABC*	3.003 ± 0.190*bcB*	
SMIG	3446.167 ± 6.113*cC*	11.433 ± 1.097*bcABC*	3.318 ± 0.320*bcB*	16.9%
SMIM	3368.306 ± 8.314*dD*	9.567 ± 1.966*cdBC*	2.839 ± 0.578*bcB*	

*Data on water consumption, dry weight (including above- and below-ground biomass), and water-use efficiency are based on the average value of 3 replicates ± standard deviation, i.e., means of three replicates ± standard errors. Means in the same column followed by different lowercase letters (a, b, c, d, e, f) are significantly different (p < 0.05); different uppercase letters (A, B, C, D, E, F) indicate highly significant differences (p < 0.01). The meaning of the “treatment condition codes SRIG, SRIM, SYIG, SYIM, SMIG and SMIM” is detailed in the notes with Figure 5.*

The results showed the following: (1) The measurements for SRIG and SRIM did not differ significantly in terms of dry matter weight (*P* > 0.05). On the other hand, the results for these two treatment conditions differed very significantly in terms of water consumption of the pepper crop (P < 0.01), and significantly in terms of WUE (*P* < 0.05). In particular, the WUE for SRIG was higher than that for SRIM by approximately 26.7%. This indicates that the irrigation mode strongly affected the WUE and, specifically, the water consumption of the SR. IG significantly reduced the water loss and thus considerably improved the WUE. (2) The measurements for SYIG (SMIG) and SYIM (SMIM) did not differ significantly in terms of the WUE (*P* > 0.05). This indicates that the effect of irrigation types on WUE was not significant in SY and SM, which is consistent with the results of the significance analysis of irrigation types on WUE (*P* = 0.983 > 0.05) under the overall sample in Table 3. (3) As can be seen from the multiple comparisons in Table 5, only the treatments under SR showed significant or highly significant differences compared to the treatments under SY and SM, and as shown by the ANOVA in Table 3, the effect of soil type on WUE was highly significant (*P* = 0.006 < 0.01). Therefore, further multiple comparisons of all six destructive sampling samples are required for a more complete understanding of the effect of soil type on WUE at each time period.

**FIGURE 7:**
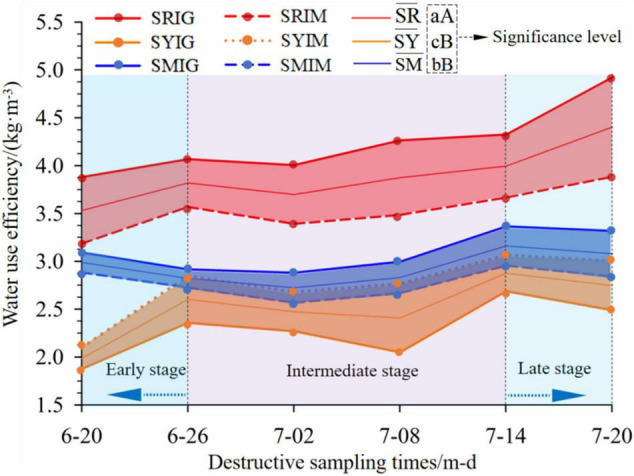
Dynamic plot of WUE under six destructive samplings during the whole growth cycle of pepper. $\bar{\text{SR}}$, $\bar{\text{SY}}$, and $\bar{\text{SM}}$ represent the mean lines of water-use efficiency under two irrigation methods in red soil, yellow sandy soil, and mixed soil in Yunnan, respectively. Letters indicate significance levels; different lowercase letters (a, b, c) in the same column indicate significant differences (*p* < 0.05), whereas different capital letters (A, B) in the same column indicate highly significant differences (*p* < 0.01). The meaning of the “treatment condition codes SRIG, SRIM, SYIG, SYIM, SMIG and SMIM” is detailed in the notes with Figure 5.

To further determine the dynamic variations in the WUE of the pepper crop throughout its growth period, the WUEs of the pepper crop based on six destructive samplings were analyzed throughout its growth period (Figure 7), and the significant effect of soil type on WUE was further analyzed.

The results showed the following: (1) The WUE for the SR exhibited an overall increasing trend. The WUEs of the six replicates for SRIG were higher than those for SRIM. The WUE for SRIG increased at significantly higher rates during the late period (from July 14 to July 20), whereas that for SRIM increased at an approximately constant rate from the middle period (July 2) to the time of berry harvesting. (2) For the SY, the SYIG and SYIM treatment conditions resulted in consistent WUE variations, that is, increasing in the early period, decreasing and then increasing in the middle period, and decreasing in the late period. The WUEs of the six replicates for SYIG were lower than those for SYIM. (3) For the SM, the SMIG and SMIM treatment conditions also resulted in largely consistent WUE variations, that is, decreasing and then increasing in the early-to-middle period and decreasing in the late period. The WUEs of the six replicates for SMIG were higher than those for SMIM. (4) From the middle period (June 26) to the time of berry harvesting (July 20), the SYIM and SMIM treatment conditions resulted in overlapping area diagrams, and the results for the two treatment conditions exhibited very consistent WUE variation trends. (5) Based on the average WUEs for the three tested soils subjected to the two irrigation modes, the soils differed significantly in terms of the average WUE between the two irrigation modes (IG and IM)—the difference between $\bar{\text{SR}}$ and $\bar{\text{SY}}$ and that between $\bar{\text{SR}}$ and $\bar{\text{SM}}$ were very significant (*P* < 0.01), whereas the difference between $\bar{\text{SY}}$ and $\bar{\text{SM}}$ was significant (*P* < 0.05). That is, as the soil clay content increased (SR > SM > SY), the WUE increased ($\bar{\text{SR}}$ > $\bar{\text{SM}}$ > $\bar{\text{SY}}$).

## Discussion

This study investigated a small-scale soil-water environment for potted crop cultivation. The difference is that our study used an in-house-designed device for measuring water infiltration, a closed-loop method was developed for calculating the water infiltration in a small-scale soil–water environment for potted crop cultivation based on the principle of water conservation. This method was consistent with the experimental principle used by Zhang et al. (2019a) to investigate the water consumption of bok choy on Yunnan laterite under IG in potted cultivation. The difference is that this study used an improved soil water content measurement method. Zhang et al. (2019a) measured directly water content using a moisture meter, this study introduces a correction coefficient K to correct measurements obtained using the moisture meter. This improved method provided more accurate quantitative calculations. However, the potted soil mass was defined as consisting of three layers for water content measurement, such that the water content within each layer was assumed to be uniform, that is, the heterogeneity of water distribution within individual layers was neglected. In future research, the gradient-change characteristic of soil water distribution must be considered to fine-tune the soil zoning for water content measurement and provide more accurate measurement data.

Numerous factors such as meteorological factors, soil characteristics, irrigation modes, and irrigation schemes affect soil water content variation. This study attempted to understand the variations in the water content of the SR in the absence of crop cultivation for different irrigation modes, with the aim of providing a basis for the subsequent investigation of crop WUE. Yu et al. (2017) investigated the water content variations of a Yunnan red loam (compared with those of a sandy loam and sand) under micro-irrigation and determined that the soil type significantly affected the soil water content. However, they did not report on whether the irrigation mode–soil type interaction had a significant effect on soil water-content variation. In contrast, this study investigated two typical irrigation modes (IM and IG) and quantitatively demonstrated that the irrigation mode–soil type interaction had a very significant effect on soil evaporation. The SR (compared to the SY and SM) had a higher water content and lower evaporation under IG. The above results, from the perspective of the irrigation technique, were because IG uses emitters buried below the soil surface, with the discharged water concentrated in the root zone and a low water content in the surface soil layer, thus effectively inhibiting water loss by evaporation. In addition, from the perspective of soil physical properties, the SR has a higher clay content, minimal hydraulic conductivity, larger surface area, and the SR pore structure (Yu et al., 2017) has a high content of clay and silt (>70%), a large number of small particle surface pores, thus, a large capacity for water retention and absorption. This research clarifies a series of soil water variation rules under the irrigation mode–soil type interaction, but the influence mechanisms of different soil physical property parameters on soil water content variation are yet to be researched in depth.

Liu et al. (2021) investigated the effect of gravel content on the water transport characteristics of a red loam by mixing different proportions (0, 5, 10, and 20%) of gravel into the red loam and determined that the cumulative evaporation of the red loam increased with respect to the gravel content. In this study, the effect of the 1:1 SY–SR mixture was similar to that of the gravel–red loam mixture. The mixture of the large sand particles of SY (accounting for a proportion of 70.2%) with the SR resulted in large pores, which increased the evaporation loss to a certain degree. However, from the perspective of soil improvement, mixing a certain proportion of the SY with the SR is effective for improving the easy hardening and no uniform water distribution characteristics of the SR to a certain degree. In future research, the SR and SY can be mixed in varying proportions to investigate the evaporation response of the soil mixture to IG, thereby providing theoretical support for improving the soil used for potted crop cultivation.

The improvement of the crop WUE is a critical problem in achieving high-efficiency use of agricultural water. Zhang et al. (2019a) determined that the WUE of bok choy cultivated under IG was higher than that under IM by approximately 24%. They also investigated Yunnan red loam used in potted crop cultivation and applied a similar irrigation scheme and a similar irrigation mode. However, IG demonstrated markedly different water-saving potentials in the cultivation of bok choy and pepper (the mean values of WUE under six destructive sampling, the WUE of bok choy was higher than that of pepper by approximately 20%). This suggests that IG has a larger water-saving potential for leafy vegetables than for solanaceous vegetables. In future research, the water-saving potentials under IG can be compared between leafy, solanaceous, and root and stem vegetables. On this basis, customized IG solutions can be designed for different crops based on their different physiological characteristics.

As shown in Figure 7, the WUE for the SR was markedly higher than those for the SY and SM under both irrigation modes, and the WUE in the late stage of crop growth exhibited accelerated increases, particularly under IG. In contrast, the SY and SM exhibited these properties in a decreasing trend. This result may be related to the different water and fertility retention capacities of soils, and root activity (Bossio et al., 2010; Chen et al., 2018). The SR is a clay and has a better water retention capacity and higher nutrient level than the SY and SM. In addition, IG transports water to the root zone more precisely, thus improving the root activity and leading to an even higher WUE for the pepper crop in the late stage of its growth. Figure 7 also shows that the average WUE for the SM was 1.84 times higher than that for the SY. This raises a question: is there an SY–SR ratio that can significantly improve the WUE to a level higher than that for pure SR? This question is subject to future experimental investigations.

## Conclusion

In the absence of crop cultivation, the soil water content and infiltration loss for all combinations of treatment conditions that were tested in this study first increased rapidly and then decreased slowly, indicating that these two parameters varied in the same direction. The Yunnan red soil subjected to root-zone micro-irrigation (SRIG) is the most desirable set of conditions for soil water retention. The variations in soil evaporation in the absence of crop cultivation can be expressed using a cubic polynomial function. Among the soils that were tested, the Yunnan red loam soil (SR) had the smallest soil evaporation, followed by the Yunnan red loam–yellow sand mixed soil (SM) and yellow sand soil (SY).

During crop (pepper) cultivation, the Yunnan red loam soil had the most stable water content under root-zone micro-irrigation (IG), whereas the Yunnan red loam–yellow sand mixed soil had the smallest water content variations under surface-drip irrigation (IM). The pepper average water-use efficiency (WUE) under root-zone micro-irrigation was about 20.2% higher than that under surface-drip irrigation in the Yunnan red loam soil. So, for Yunnan red loam soil, root-zone micro-irrigation has higher water saving efficiency compared to surface-drip irrigation, while in the yellow sand soil, the pepper average water-use efficiency under root-zone micro-irrigation was about 16.6% lower than that under surface-drip irrigation. Therefore, for yellow sand soil, surface-drip irrigation was more suitable. The comparison of yellow sand soil, Yunnan red loam–yellow sand mixed soil and Yunnan red loam soil showed that water-use efficiency increases as the soil clay content increases.

## Data Availability Statement

The original contributions presented in this study are included in the article/supplementary material, further inquiries can be directed to the corresponding author.

## Author Contributions

ZY and YY: conceptualization, validation, and writing—review and editing. ZY: methodology, funding acquisition, and resources. YQZ: software. HY: formal analysis. LK: investigation. YQZ and LK: data curation. ZY and YJZ: writing—original draft preparation. All authors have read and agreed to the published version of the manuscript.

## Conflict of Interest

The authors declare that the research was conducted in the absence of any commercial or financial relationships that could be construed as a potential conflict of interest.

## Publisher’s Note

All claims expressed in this article are solely those of the authors and do not necessarily represent those of their affiliated organizations, or those of the publisher, the editors and the reviewers. Any product that may be evaluated in this article, or claim that may be made by its manufacturer, is not guaranteed or endorsed by the publisher.
